# Summary of the Standards, Options and Recommendations for nutritional support in patients undergoing bone marrow transplantation (2002)

**DOI:** 10.1038/sj.bjc.6601091

**Published:** 2003-08-15

**Authors:** B Raynard, G Nitenberg, G Gory-Delabaere, J H Bourhis, P Bachmann, R J Bensadoun, J C Desport, D Kere, S Schneider, P Senesse, P Bordigoni, L Dieu

**Affiliations:** 1 Hôpital Antoine Béclère, Clamart, France; 2 Institut Gustave Roussy, Villejuif, France; 3 FNCLCC, Paris, France; 4 Centre Léon Bérard, Lyon, France; 5 Centre Antoine Lacassagne, Nice, France; 6 CHU Dupuytren, Limoges, France; 7 Centre Val d'Aurelle, Montpellier, France; 8 Hôpital de l'Archet, Nice, France; 9 Hôpital d'enfants de Brabois, Vandoeuvre-les-Nancy, France

**Keywords:** bone marrow transplantation, nutritional support, practice guideline

Haematological malignancy is the major indication for allogenic and autogenic bone marrow transplantation. Patients with certain solid tumours (e.g. small-cell lung cancer, breast or ovarian adenocarcinoma, germ cell tumours) may also undergo autografts, but this is usually in the setting of a clinical trial. The prognosis after bone marrow transplantation has been substantially improved by progress made in the management of infectious complications and immunosuppression (Rowe *et al*, 1994). Unfortunately, many of the drugs used (antibiotics, cyclosporine, etc.) have adverse effects, particularly metabolic and gastrointestinal effects. This is in addition to the side-effects of the conditioning regimen (total body irradiation, myelosuppressive chemotherapy), any graft complications (graft-versus-host (GVH) disease and veno-occlusive disease) and disease response (tumour lysis syndrome). Gastrointestinal and infectious complications are frequent and often lead to a significant reduction in oral food intake. In this situation, a protein-calorie deficiency can occur rapidly and this may continue even after recovery of the bone marrow.

## OBJECTIVES

The objective of these guidelines is to define the role of enteral and parenteral nutrition in the nutritional management of patients who have undergone or who will undergo bone marrow transplantation. The role of some specific substrates and emulsions (e.g. lipids, glutamine) in this clinical situation will also be considered.

There are several areas of controversy, for example, the role of glutamine supplementation (Ribaud *et al*, 1997). The importance and best means of nutritional management in patients who have undergone or are about to undergo bone marrow transplantation were addressed.

The questions addressed by the working group were:

- What are the clinical and metabolic consequences of malnutrition?
- What factors should be taken into consideration in the nutritional assessment of these patients?
- What is the efficacy of artificial nutrition (enteral or parenteral) in terms of survival?
- What is the impact on risks and infectious complications?
- What nutritional support should be used (enteral or parenteral nutrition)?
- What protein and lipid supplements should be used?
- What therapeutic strategies should be adopted?

## METHODS

Full details of the methodology used have been published (Fervers *et al*, 2001). A multidisciplinary working group was set up by the French National Federation of Comprehensive Cancer Centres (Fédération Nationale des Centres de Lutte Contre le Cancer – FNCLCC) to review the best available evidence concerning artificial nutrition in patients undergoing bone marrow transplantation. A literature search of *Medline*® from 1991 to 2001 and The *Cochrane Library*® (2001, issue 1) was performed. The search was limited to articles published in English and French. Editorials, letters, case reports and animal studies were eliminated by the search strategy. The key words were bone marrow transplantation or haematopoietic stem cell transplantation combined with nutrition or nutrition assessment. A specific search for randomised clinical trials was performed. Economic studies on this subject were not taken into consideration. The resulting reference list was completed with references from the personal reference databases of the members of the working group, including one reference published in Spanish.

Infection is the principle risk during bone marrow transplantation. Thus, the primary outcomes for evaluating the efficacy of nutritional support in patients undergoing bone marrow transplantation are the reduction in number and type of clinical infections and survival.

The secondary outcomes are:

- reduction in the length of hospitalisation in a bone marrow transplantation unit;
- reduction of the incidence and severity of the GVH reactions;
- antibiotic use;
- reduction in the volume of red blood cell and platelet transfusions;
- improved anthropometrical and laboratory parameters;
- time to the resolution of bone marrow suppression.

After selection and critical appraisal of the articles, the working group developed the ‘Standards’, ‘Options’ and ‘Recommendations’ (SORs) for nutrition support in bone marrow transplant patients based on the best available scientific evidence or on expert agreement. These guidelines were then reviewed by a group of independent experts (see the Appendix) and finalised after taking into consideration their comments.

‘*Standards*’ identify clinical situations for which there exist strong indications or contraindications for a particular intervention, and ‘*Options*’ identify situations for which there are several alternatives, none of which have shown clear superiority over the others (Table 1

**Table 1:** Definition of ‘Standards, Options and Recommendations’

Standards	Procedures or treatments that are considered to be of benefit, inappropriate or harmful by unanimous decision, based on the best available evidence
Options	Procedures or treatments that are considered to be of benefit, inappropriate or harmful by a majority, based on the best available evidence
Recommendations	Additional information to enable the available options to be ranked using explicit criteria (e.g. survival toxicity) with an indication of the level of evidence

). In any SOR, there can be several ‘*Options*’ for a given clinical situation. ‘*Recommendations*’ enable the ‘*Options*’ to be weighted according to the available evidence. Several interventions can be recommended for the same clinical situation, so that clinicians can make a choice according to specific clinical parameters, for example, local circumstances, skills, equipment, resources and patient preferences. The adaptation of the SOR to the local situation is allowable if the reason for the choice is sufficiently transparent and this is crucial for successful implementation. Inclusion of patients in clinical trials is an appropriate form of patient management in oncology and is recommended frequently within the SORs, particularly in situations where evidence is too weak to support an intervention.

The type of evidence underlying any ‘*Standard*’, ‘*Option*’ or ‘*Recommendation*’, is indicated using a classification developed by the Fédération Nationale des Centres de Lutte Contre le Cancer (FNCLCC) based on previously published models. The level of evidence depends not only on the type and quality of the studies reviewed, but also on the concordance of the results (Table 2

**Table 2:** Definition of level of evidence

*Level A*
There exists a high-standard meta-analysis or several high-standard randomised clinical trials, which give consistent results

*Level B*
There exist good quality evidence from randomised trials (B1) or prospective or retrospective studies (B2). The results are consistent when considered together

*Level C*
The methodology of the available studies is weak or their results are not consistent when considered together

*Level D*
Either the scientific data do not exist or there is only a series of cases

*Expert agreement*
The data do not exist for the method concerned, but the experts are unanimous in their judgement

). When no clear scientific evidence exists, judgement is made according to the professional experience and consensus of the working group (‘expert agreement’).

These guidelines will be updated when new scientific evidence becomes available or when there is a change in expert agreement. The French full text version has been published (Raynard *et al*, 2002) and is also available at: (http://www.fnclcc.fr). A French summary version was produced, based on the full text, and this has been translated into English here.

### Clinical and metabolic consequences of malnutrition

Moderate malnutrition is frequent before bone marrow transplantation (level of evidence: C). The prevalence of severe malnutrition before bone marrow transplantation has not been accurately assessed. Malnutrition is an independent risk factor for mortality after bone marrow transplantation (level of evidence: C).

Severe malnutrition occurs rapidly in the absence of appropriate nutritional support after myeloablative conditioning (level of evidence: C). Gut disorders are the chief cause of malnutrition and deficiency syndromes (level of evidence: C). Nitrogen deficiency is often severe after myeloablative conditioning (level of evidence: B2). A precise evaluation of the incidence of malnutrition in patients who have undergone bone marrow transplantation is necessary (recommendation, expert agreement).

### Nutritional assessment and follow-up (Figure 1)

**Figure 1:**
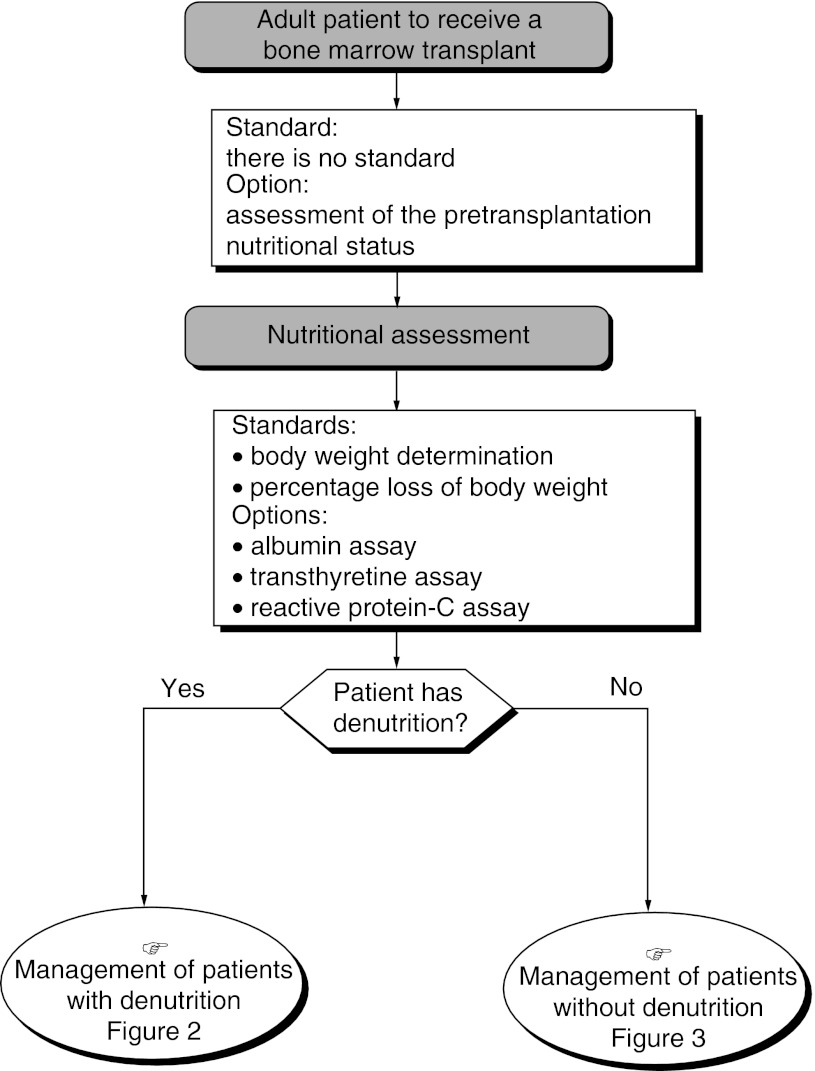
Assessment of the pretransplantation nutritional status.

There is no standard. Nutritional assessment and follow-up can be considered in patients who have undergone or are about to undergo bone marrow transplantation (option). Routine nutritional evaluation should be carried out before transplantation (recommendation, expert agreement).

### Modalities for nutritional assessment and follow-up and for assessing protein-energy intake

The initial nutritional assessment should include a body weight measurement and percentage loss of body weight (standard, expert agreement). The initial nutritional evaluation can include assays for albumin, transthyretin and C-reactive protein (option).

**Figure 2:**
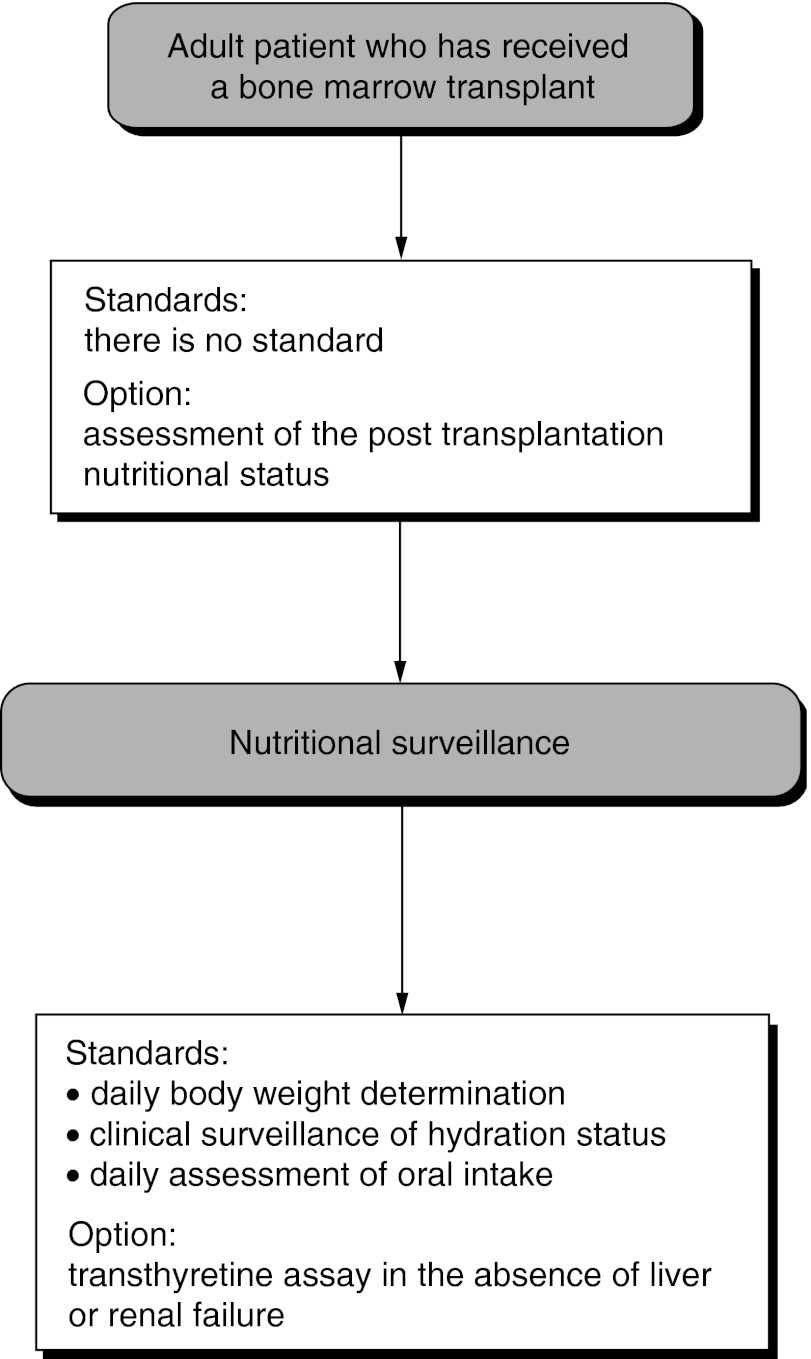
Nutritional surveillance.

Nutritional follow-up (Figure 2

) should include weekly determination of body weight, clinical surveillance of the hydration status and weekly evaluation of oral food intake (standard, expert agreement). Nutritional surveillance can include an assay for transthyretin in the absence of liver and/or kidney failure (option).

The recommended daily nonprotein calorie intake is between 25 and 35 kcal kg^−1^ and the recommended daily nitrogen intake is between 200 and 250 mg kg^−1^ (recommendation, expert agreement).

### Artificial nutrition

**Figure 3:**
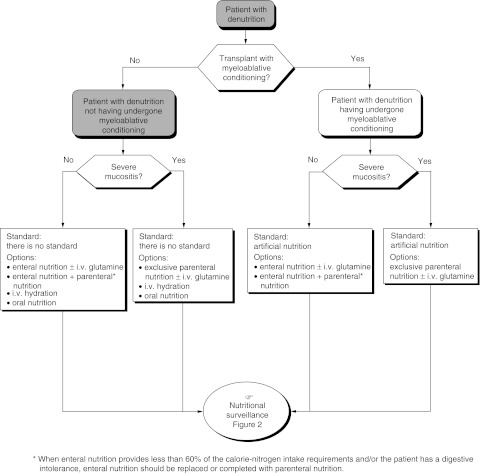
Management of patients with denutrition detected during pretransplantation assessment.

Artificial nutrition is indicated in patients after bone marrow transplantation with myeloablative conditioning (standard) (Figures 3

and 4

**Figure 4:**
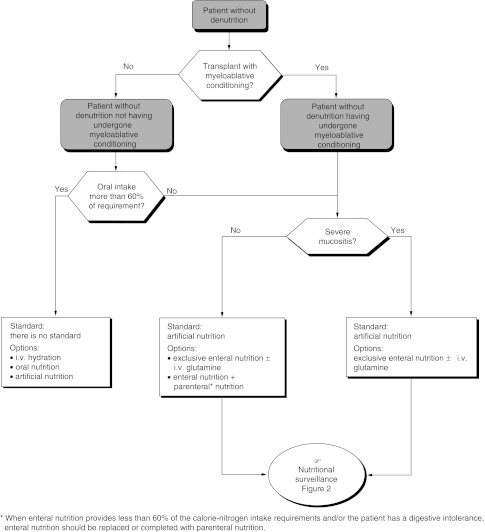
Management of patients with no denutrition detected during pretransplantation assessment

). In other situations, there is no routine indication for artificial nutrition. The different modalities of nutritional support available are artificial nutrition, intravenous hydration and oral nutrition.

The recommendations depend on the patients' initial nutritional status and the expected duration of any gastrointestinal problems. In patients who are not suffering from malnutrition after nonmyeloablative conditioning and whose oral intake is more than 60% of the requirements, oral nutrition should be maintained with intravenous hydration during hospitalisation (recommendation, expert agreement).

In patients who are not suffering from malnutrition after myeloablative conditioning, artificial nutrition should be started from day 1 (recommendation, expert agreement).

Artificial nutrition should be given to patients suffering from malnutrition (more than 10% loss of body weight) irrespective of the type of transplant or conditioning (recommendation, expert agreement).

### Oral or parenteral nutrition

There are no standard modalities for artificial nutrition (level of evidence: B1). Total enteral or parenteral nutrition or enteral nutrition combined with parenteral nutrition can be considered (option). Enteral and parenteral nutrition give the same clinical and metabolic results (level of evidence: B1).

Enteral nutrition is theoretically possible, but its safety is variable (recommendation, level of evidence: B1). Enteral nutrition rather than parenteral nutrition should be the preferred first-line treatment in patients undergoing bone marrow transplantation without myeloablative conditioning (recommendation, expert agreement). This should be combined with parenteral nutrition when the calorie-nitrogen intake is less than 60% of the requirements (recommendation, expert agreement).

Total parenteral nutrition should be reserved for patients who have an intolerance of oral or enteral nutrition, those who have an obstruction in the digestive tract or those who have severe mucositis (recommendation, expert agreement). Dietetic follow-up should be continued for some time after the graft to facilitate resumption of oral feeding and withdrawal from artificial nutrition (recommendation, expert agreement). Randomised clinical trials should be undertaken to compare enteral and parenteral nutrition (recommendation, expert agreement).

### Lipid intake

A lipid intake of 30% of the nonprotein energy intake, should be used during bone marrow transplantation (standard, level of evidence: B1) with no preference for medium-chain or long-chain triglycerides (standard, level of evidence: B1). A lipid intake of more than 30% can be considered without increasing the risk of adverse effects (option, level of evidence: B1).

Other types of lipid emulsions should only be used in the setting of a randomised clinical trial using relevant clinical outcomes in patients undergoing bone marrow transplantation (recommendation, expert agreement). A reduction in the incidence of serious GVH reactions by a high lipid intake should be confirmed (recommendation, expert agreement).

### Protein substrates

There is no justification for supplementation with nitrogen-containing substrates or oral glutamine in the nutritional support of these patients. Parenteral glutamine supplementation can be considered (option). Nitrogen-containing substrates (glutamine, branched chain amino acids, arginine, ornithine alpha-ketoglutarate) should be evaluated in large randomised clinical trials (recommendation, expert agreement).

### Strategies for nutritional support

#### Patients with myeloablative conditioning

Artificial nutrition should be considered in patients with myeloablative conditioning (standard) (Figure 3 and Figure 4). There are no standard modalities. Enteral nutrition with or without intravenous glutamine, enteral and parenteral nutrition, total parenteral nutrition with or without intravenous glutamine can all be considered. Total parenteral nutrition is recommended for patients with severe mucositis (expert agreement). Artificial nutrition should be started on day 1 (expert agreement).

#### Patients without myeloablative conditioning

There are no standards. Artificial nutrition (enteral nutrition with or without intravenous glutamine, enteral and parenteral nutrition) intravenous hydration or oral nutrition can be considered (Figure 3 and Figure 4). Total parenteral nutrition is recommended for patients with severe mucositis (expert agreement).

## Appendix

### Reviewers

D Blaise (Institut Paoli Calmettes, Marseille), JL Bornet (Hôpital de Rangueil, Toulouse), P Bournay (Centre Léon Bérard, Lyon), C Chabannon (Institut Paoli Calmettes, Marseille), C Chambrier (Hôpital Edouard Herriot, Lyon), D Cowen (Institut Paoli Calmettes, Marseille), L Cynober (Hôtel-Dieu, Paris), J Delarue (CHU La Cavale Blanche, Brest), E Fondrinier (Centre Paul Papin, Angers), Y Lallemand (Centre Léon Bérard, Lyon), A Lortholary (Centre Paul Papin, Angers), JP Lotz (Hôpital Tenon, Paris), P Maingon (Centre Georges François Leclerc, Dijon), Y Merrouche (CHU, Saint Etienne), N Milpied (Hôpital de l'Hôtel Dieu, Nantes), F Montange (Centre Alexis Vautrin, Vandoeuvre-les-Nancy), M Simon (Centre Alexis Vautrin, Vandoeuvre-les-Nancy), AM Stoppa (Institut Paoli-Calmettes, Marseille), A Thyss (Centre Antoine Lacassagne, Nice), H Tilly (Centre Henri Becquerel, Rouen).
